# Supplementary material for: Allosteric regulation and crystallographic fragment screening of SARS-CoV-2 NSP15 endoribonuclease
Source: Nucleic Acids Res. 2023 Apr 28;51(10):5255–70. doi: 10.1093/nar/gkad314 (PMC10250223; doi:10.1093/nar/gkad314)
Supplement: gkad314_Supplemental_Files [file gkad314_supplemental_files.zip › Supplementary datasets.zip!/188/Refine_9/01-BUSTER/Cycle-5/shell.01/R_Rfree.html]

# Rxpct factors in resolution bins at cycle 0


|  |  |  |  |  |  |  |  |  |
| --- | --- | --- | --- | --- | --- | --- | --- | --- |
|  | **<d> (Å)**  Rxpct\_Work | Rxpct\_Free | Rxpct\_All | |  |  |  |  |
|  | 16.0655 | 0.3726 | 0.4614 | 0.3755 |  |
|  | 9.2754 | 0.2179 | 0.2722 | 0.2211 |  |
|  | 7.1847 | 0.1938 | 0.2361 | 0.1962 |  |
|  | 6.0722 | 0.1972 | 0.2500 | 0.1995 |  |
|  | 5.3552 | 0.1535 | 0.2695 | 0.1583 |  |
|  | 4.8439 | 0.1425 | 0.2166 | 0.1469 |  |
|  | 4.4558 | 0.1396 | 0.2038 | 0.1429 |  |
|  | 4.1481 | 0.1466 | 0.2072 | 0.1501 |  |
|  | 3.8965 | 0.1542 | 0.2335 | 0.1579 |  |
|  | 3.6857 | 0.1639 | 0.2524 | 0.1679 |  |
|  | 3.5058 | 0.1708 | 0.2085 | 0.1727 |  |
|  | 3.3499 | 0.1772 | 0.2358 | 0.1805 |  |
|  | 3.2131 | 0.1925 | 0.2196 | 0.1941 |  |
|  | 3.0918 | 0.1929 | 0.2651 | 0.1962 |  |
|  | 2.9833 | 0.1972 | 0.2365 | 0.1988 |  |
|  | 2.8855 | 0.2046 | 0.2443 | 0.2066 |  |
|  | 2.7967 | 0.2057 | 0.2588 | 0.2081 |  |
|  | 2.7156 | 0.2127 | 0.2499 | 0.2145 |  |
|  | 2.6412 | 0.2631 | 0.2630 | 0.2631 |  |
|  | 2.5725 | 0.3535 | 0.2710 | 0.3508 |  |
|  | **Overall** | 0.1835 | 0.2413 | 0.1864 |  |

# Rxpct factors in resolution bins at cycle 1


|  |  |  |  |  |  |  |  |  |
| --- | --- | --- | --- | --- | --- | --- | --- | --- |
|  | **<d> (Å)**  Rxpct\_Work | Rxpct\_Free | Rxpct\_All | |  |  |  |  |
|  | 16.0655 | 0.3728 | 0.4607 | 0.3757 |  |
|  | 9.2754 | 0.2177 | 0.2724 | 0.2209 |  |
|  | 7.1847 | 0.1939 | 0.2378 | 0.1964 |  |
|  | 6.0722 | 0.1974 | 0.2516 | 0.1998 |  |
|  | 5.3552 | 0.1531 | 0.2698 | 0.1579 |  |
|  | 4.8439 | 0.1425 | 0.2165 | 0.1468 |  |
|  | 4.4558 | 0.1395 | 0.2040 | 0.1428 |  |
|  | 4.1481 | 0.1466 | 0.2075 | 0.1501 |  |
|  | 3.8965 | 0.1542 | 0.2337 | 0.1579 |  |
|  | 3.6857 | 0.1639 | 0.2526 | 0.1679 |  |
|  | 3.5058 | 0.1708 | 0.2087 | 0.1727 |  |
|  | 3.3499 | 0.1771 | 0.2358 | 0.1805 |  |
|  | 3.2131 | 0.1925 | 0.2196 | 0.1941 |  |
|  | 3.0918 | 0.1929 | 0.2650 | 0.1961 |  |
|  | 2.9833 | 0.1971 | 0.2364 | 0.1988 |  |
|  | 2.8855 | 0.2045 | 0.2442 | 0.2066 |  |
|  | 2.7967 | 0.2057 | 0.2587 | 0.2080 |  |
|  | 2.7156 | 0.2126 | 0.2498 | 0.2144 |  |
|  | 2.6412 | 0.2630 | 0.2628 | 0.2630 |  |
|  | 2.5725 | 0.3534 | 0.2712 | 0.3507 |  |
|  | **Overall** | 0.1834 | 0.2415 | 0.1864 |  |

# Rxpct factors in resolution bins at cycle 2


|  |  |  |  |  |  |  |  |  |
| --- | --- | --- | --- | --- | --- | --- | --- | --- |
|  | **<d> (Å)**  Rxpct\_Work | Rxpct\_Free | Rxpct\_All | |  |  |  |  |
|  | 16.0655 | 0.3728 | 0.4607 | 0.3757 |  |
|  | 9.2754 | 0.2177 | 0.2724 | 0.2209 |  |
|  | 7.1847 | 0.1939 | 0.2377 | 0.1964 |  |
|  | 6.0722 | 0.1974 | 0.2517 | 0.1998 |  |
|  | 5.3552 | 0.1531 | 0.2698 | 0.1579 |  |
|  | 4.8439 | 0.1425 | 0.2164 | 0.1468 |  |
|  | 4.4558 | 0.1395 | 0.2040 | 0.1429 |  |
|  | 4.1481 | 0.1466 | 0.2075 | 0.1502 |  |
|  | 3.8965 | 0.1542 | 0.2337 | 0.1580 |  |
|  | 3.6857 | 0.1640 | 0.2526 | 0.1679 |  |
|  | 3.5058 | 0.1709 | 0.2088 | 0.1727 |  |
|  | 3.3499 | 0.1771 | 0.2358 | 0.1805 |  |
|  | 3.2131 | 0.1925 | 0.2195 | 0.1941 |  |
|  | 3.0918 | 0.1929 | 0.2649 | 0.1961 |  |
|  | 2.9833 | 0.1971 | 0.2363 | 0.1987 |  |
|  | 2.8855 | 0.2045 | 0.2441 | 0.2065 |  |
|  | 2.7967 | 0.2057 | 0.2586 | 0.2080 |  |
|  | 2.7156 | 0.2126 | 0.2498 | 0.2144 |  |
|  | 2.6412 | 0.2630 | 0.2628 | 0.2630 |  |
|  | 2.5725 | 0.3534 | 0.2712 | 0.3507 |  |
|  | **Overall** | 0.1834 | 0.2415 | 0.1864 |  |

# Rxpct factors in resolution bins at cycle 3


|  |  |  |  |  |  |  |  |  |
| --- | --- | --- | --- | --- | --- | --- | --- | --- |
|  | **<d> (Å)**  Rxpct\_Work | Rxpct\_Free | Rxpct\_All | |  |  |  |  |
|  | 16.0655 | 0.3691 | 0.4669 | 0.3723 |  |
|  | 9.2754 | 0.2133 | 0.2693 | 0.2166 |  |
|  | 7.1847 | 0.1910 | 0.2411 | 0.1938 |  |
|  | 6.0722 | 0.1944 | 0.2535 | 0.1970 |  |
|  | 5.3552 | 0.1516 | 0.2704 | 0.1565 |  |
|  | 4.8439 | 0.1419 | 0.2128 | 0.1461 |  |
|  | 4.4558 | 0.1397 | 0.2070 | 0.1432 |  |
|  | 4.1481 | 0.1455 | 0.2048 | 0.1490 |  |
|  | 3.8965 | 0.1539 | 0.2329 | 0.1576 |  |
|  | 3.6857 | 0.1644 | 0.2537 | 0.1684 |  |
|  | 3.5058 | 0.1712 | 0.2148 | 0.1733 |  |
|  | 3.3499 | 0.1781 | 0.2346 | 0.1813 |  |
|  | 3.2131 | 0.1925 | 0.2137 | 0.1937 |  |
|  | 3.0918 | 0.1931 | 0.2601 | 0.1961 |  |
|  | 2.9833 | 0.1968 | 0.2337 | 0.1984 |  |
|  | 2.8855 | 0.2036 | 0.2425 | 0.2056 |  |
|  | 2.7967 | 0.2055 | 0.2557 | 0.2077 |  |
|  | 2.7156 | 0.2112 | 0.2500 | 0.2131 |  |
|  | 2.6412 | 0.2588 | 0.2673 | 0.2594 |  |
|  | 2.5725 | 0.3534 | 0.2893 | 0.3513 |  |
|  | **Overall** | 0.1824 | 0.2410 | 0.1854 |  |
